# Supplementary material for: HIVprotI: an integrated web based platform for prediction and design of HIV proteins inhibitors
Source: J Cheminform. 2018 Mar 9;10:12. doi: 10.1186/s13321-018-0266-y (PMC5845081; doi:10.1186/s13321-018-0266-y)
Supplement: Supplementary file 2 — Additional file 2. Source code of HIVProtI web server. [file 13321_2018_266_MOESM2_ESM.zip › HIVprotI_Source-code/hivprotI/weka-3-6-6/doc/weka/core/neighboursearch/NearestNeighbourSearch.html]

NearestNeighbourSearch


---


|  |  |  |
| --- | --- | --- |
| **Overview** \| **Package** \| **Class** \| **Tree** \| **Deprecated** \| **Index** \| **Help** \| **Weka's home** | |  |
| **PREV CLASS**   **NEXT CLASS** | **FRAMES**    **NO FRAMES**     **All Classes** |  |
| SUMMARY: NESTED \| FIELD \| CONSTR \| METHOD | DETAIL: FIELD \| CONSTR \| METHOD |  |


---


## weka.core.neighboursearch Class NearestNeighbourSearch

```
java.lang.Object
  weka.core.neighboursearch.NearestNeighbourSearch
```

**All Implemented Interfaces:**: java.io.Serializable, AdditionalMeasureProducer, OptionHandler, RevisionHandler

**Direct Known Subclasses:**: BallTree, CoverTree, KDTree, LinearNNSearch

---

``` public abstract class NearestNeighbourSearch extends java.lang.Object implements java.io.Serializable, OptionHandler, AdditionalMeasureProducer, RevisionHandler ```

Abstract class for nearest neighbour search. All algorithms (classes) that
do nearest neighbour search should extend this class.

**Version:**
:   $Revision: 1.2 $

**Author:**
:   Ashraf M. Kibriya (amk14[at-the-rate]cs[dot]waikato[dot]ac[dot]nz)

**See Also:**: Serialized Form

---

**Constructor Summary**

`NearestNeighbourSearch()`
  
          Constructor.

`NearestNeighbourSearch(Instances insts)`
  
          Constructor.


| **Method Summary** | |
| --- | --- |
| `void` | `addInstanceInfo(Instance ins)`             Adds information from the given instance without modifying the datastructure a lot. |
| `static void` | `combSort11(double[] arrayToSort, int[] linkedArray)`             sorts the two given arrays. |
| `java.lang.String` | `distanceFunctionTipText()`             Returns the tip text for this property. |
| `java.util.Enumeration` | `enumerateMeasures()`             Returns an enumeration of the additional measure names. |
| `DistanceFunction` | `getDistanceFunction()`             returns the distance function currently in use. |
| `abstract  double[]` | `getDistances()`             Returns the distances of the k nearest neighbours. |
| `Instances` | `getInstances()`             returns the instances currently set. |
| `double` | `getMeasure(java.lang.String additionalMeasureName)`             Returns the value of the named measure. |
| `boolean` | `getMeasurePerformance()`             Gets whether performance statistics are being calculated or not. |
| `java.lang.String[]` | `getOptions()`             Gets the current settings. |
| `PerformanceStats` | `getPerformanceStats()`             Gets the class object that contains the performance statistics of the search method. |
| `java.lang.String` | `globalInfo()`             Returns a string describing this nearest neighbour search algorithm. |
| `abstract  Instances` | `kNearestNeighbours(Instance target, int k)`             Returns k nearest instances in the current neighbourhood to the supplied instance. |
| `java.util.Enumeration` | `listOptions()`             Returns an enumeration describing the available options. |
| `java.lang.String` | `measurePerformanceTipText()`             Returns the tip text for this property. |
| `abstract  Instance` | `nearestNeighbour(Instance target)`             Returns the nearest instance in the current neighbourhood to the supplied instance. |
| `static void` | `quickSort(double[] arrayToSort, double[] linkedArray, int left, int right)`             performs quicksort. |
| `void` | `setDistanceFunction(DistanceFunction df)`             sets the distance function to use for nearest neighbour search. |
| `void` | `setInstances(Instances insts)`             Sets the instances. |
| `void` | `setMeasurePerformance(boolean measurePerformance)`             Sets whether to calculate the performance statistics or not. |
| `void` | `setOptions(java.lang.String[] options)`             Parses a given list of options. |
| `abstract  void` | `update(Instance ins)`             Updates the NearNeighbourSearch algorithm for the new added instance. |

**Methods inherited from class java.lang.Object**

`equals, getClass, hashCode, notify, notifyAll, toString, wait, wait, wait`

**Methods inherited from interface weka.core.RevisionHandler**

`getRevision`

**Constructor Detail**

### NearestNeighbourSearch

```
public NearestNeighbourSearch()
```

:   Constructor.

---


### NearestNeighbourSearch

```
public NearestNeighbourSearch(Instances insts)
```

:   Constructor.

    **Parameters:**: `insts` - The set of instances that constitute the neighbourhood.


**Method Detail**

### globalInfo

```
public java.lang.String globalInfo()
```

:   Returns a string describing this nearest neighbour search algorithm.

    :   **Returns:**: a description of the algorithm for displaying in the explorer/experimenter gui

---


### listOptions

```
public java.util.Enumeration listOptions()
```

:   Returns an enumeration describing the available options.

    :   **Specified by:**: `listOptions` in interface `OptionHandler`
    :   **Returns:**: an enumeration of all the available options.

---


### setOptions

```
public void setOptions(java.lang.String[] options)
                throws java.lang.Exception
```

:   Parses a given list of options. Valid options are:


    :   **Specified by:**: `setOptions` in interface `OptionHandler`
    :   **Parameters:**: `options` - the list of options as an array of strings **Throws:**: `java.lang.Exception` - if an option is not supported

---


### getOptions

```
public java.lang.String[] getOptions()
```

:   Gets the current settings.

    :   **Specified by:**: `getOptions` in interface `OptionHandler`
    :   **Returns:**: an array of strings suitable for passing to setOptions()

---


### distanceFunctionTipText

```
public java.lang.String distanceFunctionTipText()
```

:   Returns the tip text for this property.

    :   **Returns:**: tip text for this property suitable for displaying in the explorer/experimenter gui

---


### getDistanceFunction

```
public DistanceFunction getDistanceFunction()
```

:   returns the distance function currently in use.

    :   **Returns:**: the distance function

---


### setDistanceFunction

```
public void setDistanceFunction(DistanceFunction df)
                         throws java.lang.Exception
```

:   sets the distance function to use for nearest neighbour search.

    :   **Parameters:**: `df` - the new distance function to use **Throws:**: `java.lang.Exception` - if instances cannot be processed

---


### measurePerformanceTipText

```
public java.lang.String measurePerformanceTipText()
```

:   Returns the tip text for this property.

    :   **Returns:**: tip text for this property suitable for displaying in the explorer/experimenter gui

---


### getMeasurePerformance

```
public boolean getMeasurePerformance()
```

:   Gets whether performance statistics are being calculated or not.

    :   **Returns:**: true if the measure performance is calculated

---


### setMeasurePerformance

```
public void setMeasurePerformance(boolean measurePerformance)
```

:   Sets whether to calculate the performance statistics or not.

    :   **Parameters:**: `measurePerformance` - if true then the performance is calculated

---


### nearestNeighbour

```
public abstract Instance nearestNeighbour(Instance target)
                                   throws java.lang.Exception
```

:   Returns the nearest instance in the current neighbourhood to the supplied
    instance.

    :   **Parameters:**: `target` - The instance to find the nearest neighbour for. **Returns:**: the nearest neighbor **Throws:**: `java.lang.Exception` - if the nearest neighbour could not be found.

---


### kNearestNeighbours

```
public abstract Instances kNearestNeighbours(Instance target,
                                             int k)
                                      throws java.lang.Exception
```

:   Returns k nearest instances in the current neighbourhood to the supplied
    instance.

    :   **Parameters:**: `target` - The instance to find the k nearest neighbours for.: `k` - The number of nearest neighbours to find. **Returns:**: the k nearest neighbors **Throws:**: `java.lang.Exception` - if the neighbours could not be found.

---


### getDistances

```
public abstract double[] getDistances()
                               throws java.lang.Exception
```

:   Returns the distances of the k nearest neighbours. The kNearestNeighbours
    or nearestNeighbour needs to be called first for this to work.

    :   **Returns:**: the distances **Throws:**: `java.lang.Exception` - if called before calling kNearestNeighbours or nearestNeighbours.

---


### update

```
public abstract void update(Instance ins)
                     throws java.lang.Exception
```

:   Updates the NearNeighbourSearch algorithm for the new added instance.
    P.S.: The method assumes the instance has already been added to the
    m\_Instances object by the caller.

    :   **Parameters:**: `ins` - the instance to add **Throws:**: `java.lang.Exception` - if updating fails

---


### addInstanceInfo

```
public void addInstanceInfo(Instance ins)
```

:   Adds information from the given instance without modifying the
    datastructure a lot.

    :   **Parameters:**: `ins` - the instance to add the information from

---


### setInstances

```
public void setInstances(Instances insts)
                  throws java.lang.Exception
```

:   Sets the instances.

    :   **Parameters:**: `insts` - the instances to use **Throws:**: `java.lang.Exception` - if setting fails

---


### getInstances

```
public Instances getInstances()
```

:   returns the instances currently set.

    :   **Returns:**: the current instances

---


### getPerformanceStats

```
public PerformanceStats getPerformanceStats()
```

:   Gets the class object that contains the performance statistics of
    the search method.

    :   **Returns:**: the performance statistics

---


### enumerateMeasures

```
public java.util.Enumeration enumerateMeasures()
```

:   Returns an enumeration of the additional measure names.

    :   **Specified by:**: `enumerateMeasures` in interface `AdditionalMeasureProducer`
    :   **Returns:**: an enumeration of the measure names

---


### getMeasure

```
public double getMeasure(java.lang.String additionalMeasureName)
```

:   Returns the value of the named measure.

    :   **Specified by:**: `getMeasure` in interface `AdditionalMeasureProducer`
    :   **Parameters:**: `additionalMeasureName` - the name of the measure to query for its value **Returns:**: the value of the named measure **Throws:**: `java.lang.IllegalArgumentException` - if the named measure is not supported

---


### combSort11

```
public static void combSort11(double[] arrayToSort,
                              int[] linkedArray)
```

:   sorts the two given arrays.

    :   **Parameters:**: `arrayToSort` - The array sorting should be based on.: `linkedArray` - The array that should have the same ordering as arrayToSort.

---


### quickSort

```
public static void quickSort(double[] arrayToSort,
                             double[] linkedArray,
                             int left,
                             int right)
```

:   performs quicksort.

    :   **Parameters:**: `arrayToSort` - the array to sort: `linkedArray` - the linked array: `left` - the first index of the subset: `right` - the last index of the subset


---


|  |  |  |
| --- | --- | --- |
| **Overview** \| **Package** \| **Class** \| **Tree** \| **Deprecated** \| **Index** \| **Help** \| **Weka's home** | |  |
| **PREV CLASS**   **NEXT CLASS** | **FRAMES**    **NO FRAMES**     **All Classes** |  |
| SUMMARY: NESTED \| FIELD \| CONSTR \| METHOD | DETAIL: FIELD \| CONSTR \| METHOD |  |


---
